# Investigating the relationship between autistic traits and symptoms and Catatonia Spectrum

**DOI:** 10.1192/j.eurpsy.2022.2334

**Published:** 2022-11-04

**Authors:** L. Dell’Osso, G. Amatori, G. Massimetti, B. Nardi, D. Gravina, F. Benedetti, C. Bonelli, M. Luciano, I. Berardelli, N. Brondino, M. De Gregorio, G. Deste, M. Nola, A. Reitano, M. R. A. Muscatello, M. Pompili, P. Politi, A. Vita, C. Carmassi, M. Maj

**Affiliations:** 1 Department of Clinical and Experimental Medicine, University of Pisa, Pisa, Italy; 2 Department of Psychiatry, University of Naples “Luigi Vanvitelli”, Naples, Italy; 3 Department of Neuroscience, Mental Health and Sense Organs, University of Roma “La Sapienza”, Rome, Italy; 4 Department of Brain and Behavioral Sciences, University of Pavia, Pavia, Italy; 5 Department of Biomedical and Dental Sciences and Morphofunctional Imaging, University of Messina, Messina, Italy; 6 Department of Clinical and Experimental Sciences, University of Brescia, Brescia, Italy

**Keywords:** AdAS Spectrum, autism, autism spectrum, catatonia, Catatonia Spectrum

## Abstract

**Background:**

In recent years, numerous studies have highlighted the overlap between autism spectrum disorder (ASD) and catatonia, both from a clinical and pathophysiological perspective. This study aimed to investigate the relationship between the autism spectrum (autistic traits and ASD signs, symptoms, and behavioral manifestation) and Catatonia Spectrum (CS).

**Methods:**

A total sample of 376 subjects was distributed in four diagnostic groups. Subjects were assessed with the Structured Clinical Interview for DSM-5, Research Version, the Adult Autism Subthreshold Spectrum (AdAS Spectrum), and CS. In the statistical analyses, the total sample was also divided into three groups according to the degree of autism severity, based on the AdAS Spectrum total score.

**Results:**

A statistically significant positive correlation was found between AdAS Spectrum and CS total score within the total sample, the gender subgroups, and the diagnostic categories. The AdAS Spectrum domains found to be significantly and strongly correlated with the total CS score were hyper–hypo reactivity to sensory input, verbal communication, nonverbal communication, restricted interests and rumination, and inflexibility and adherence to routine. The three groups of different autistic severity were found to be distributed across all diagnostic groups and the CS score increased significantly from the group without autistic traits to the group with ASD.

**Conclusions:**

Our study reports a strong correlation between autism spectrum and CS.

## Introduction

Over the past two decades, the relationship between autism spectrum disorder (ASD) and catatonia has been investigated by numerous studies in the scientific literature. ASDs are neurodevelopmental disorders characterized by difficulties in social communication, restricted and repetitive behaviors or interests, and hyper/hyporesponsiveness to sensory stimuli. In addition to the diagnosis of overt ASD, great importance has been given in recent years to subthreshold autistic manifestations, widespread to varying degrees in the general population and investigated through dedicated assessment tools [1]. Catatonia, originally described in 1874 by Kahlbaum and long relegated to the realm of schizophrenia, is currently defined by the DSM-5-TR [2] as a severe neuropsychiatric syndrome characterized by three or more symptoms among catalepsy, waxy flexibility, stupor, muteness, negativism, agitation, posturing, stereotypes, mannerisms, grimacing, echolalia, and echopraxia. The DSM-5-TR trajectory appeared to be receptive to a less strict definition of the catatonic clinical picture and an expansion of its boundaries. It has been stressed that the lack of narrow boundaries may result in the inclusion of overly divergent conditions, like akathisia, under the shadow of catatonic symptoms [3]. Although provocative, these critiques raise reflexive questions about the existence of something we might call a spectrum of catatonia that could recognize a wide range of etiologies and a wide range of manifestations with varying degrees of severity, which is somewhat consistent with the conceptualization of catatonia in DSM-5 as a *trans*-diagnostic specifier, potentially associated with every mental disorder, with a multitude of different combinations of symptoms, and denoting atypical and incomplete manifestations under the umbrella of catatonia not otherwise specified. Prominent among the mental disorders associated with catatonia are neurodevelopmental disorders and, among them, ASD. The complication of ASD with catatonia is not an infrequent occurrence, as already observed by Lorna Wing’s studies of autism [4]. A recent systematic review [5] showed that 10.4% of individuals affected by ASD have catatonia, highlighting a clinical overlap between the two disorders for which several explanations have been proposed: a common alteration in the GABAergic system [6], in neural circuits [7] or in the size of cerebellar structures [8], as well as a possible genetic connection, derived from susceptibility regions on chromosome 15 [9].

In two prevalence studies [4, 10], catatonia was found to occur in 12–17% of a large sample of adolescents and young adults with ASD. Numerous clinical features are shared by ASD and catatonia, such as mutism and echolalia, stereotyped movements and repetitive behaviors, and negativism and arousal. This clinical overlap could be responsible, on one hand, for a propensity to overestimate subthreshold catatonia among autistic subjects and, on the other, for a failure to recognize catatonic symptoms that first appear in patients with ASD [11]. Indeed, although anecdotal, previous descriptions have indicated that catatonia may slowly develop over the course of autism, often preceded by isolated manifestations and a slow deterioration in functioning, until it assumes a chronic character [12]. One of the few systematic studies on catatonia among young people with ASD found that the largest percentage of autism-related cases occur among individuals with Asperger’s disorder (AD), rather than among classic or atypical autistic patients [12]. As it is known that a significant percentage of patients with AD remain undiagnosed throughout adolescence and adulthood [13], it would be reasonable to consider the possibility that they may develop catatonia at some point in their lives. Such a scenario would require, from clinicians working with adults, who are often unfamiliar with the diagnosis of ASD, an ability to untangle the complex clinical picture of such patients and to identify appropriate treatment.

Moreover, growing literature supports the existence of an autistic subthreshold phenotype, free from significant impairment in daily living but phenomenologically and etiologically in continuity with the clinical spectrum of autistic disorders. Such subthreshold autism spectrum, although unrecognized, could increase the risk of catatonia. Since it is believed that the autistic dimension is not only present in individuals with ASD, but also widespread in the general population as a broader autistic phenotype or autistic traits [1], we might speculate that catatonia might be related to this autistic dimension even when not clinically evident and, as discussed in recent work, especially in patients with a history of traumatic events [14]. Beyond the recurrent speculations regarding the conceptualization of autism as an early form of catatonia, some studies conducted on a validated animal model of autism have shown the beneficial effect of pharmacologically induced seizures and electroconvulsive therapy, one of the most effective treatments available for catatonia, in reducing autism-like behaviors in mice [15]. In addition, several studies on the pathophysiology of catatonia and autism suggest the existence of a common phenomenon of GABAergic cortical dysregulation resulting in an imbalance between arousal and inhibition in the CNS and a failure to cognitively control emotions [16, 17]. In parallel, candidate loci for autism and catatonia have been identified on the long arm of chromosome 15 [18–20].

The aim of this study is to investigate the relationship between autism traits, signs, symptoms, and manifestations, and catatonic spectrum and to determine which among the autism spectrum features are the most related to the catatonic spectrum.

## Materials and Methods

Data have been collected between November 2021 and January 2022 at six Italian University Departments of Psychiatry, coordinated by the University of Pisa: University of Campania “Luigi Vanvitelli,” University of Pavia, University of Messina, La Sapienza University of Rome, and University of Brescia.

### Study sample and procedures

The total sample consisted of 376 subjects distributed in four diagnostic groups, all evaluated according to DSM-5 diagnostic criteria. Exclusion criteria were: age under 18 years, language or intellectual impairment affecting the possibility to fulfill the assessments, mental disability, poor cooperation skills, and ongoing psychotic symptoms. Specifically, the four groups were individuated as follows: 86 subjects endorsing at least three symptom criteria for catatonia (CTN); 81 subjects diagnosed with borderline personality disorder (BPD); 104 subjects diagnosed with major depressive disorder (MDD); 105 healthy control individuals without current or lifetime mental disorders (HC) and belonging to health care and paramedical personnel. All subjects were aged 18–60 years old and signed a written informed consent. The Structured Clinical Interview for DSM-5, Research Version (SCID-5-RV) [21] was used to confirm the diagnoses of BPD and MDD, as well as the absence of mental disorders among CTL. The study was conducted in accordance with the Declaration of Helsinki. The Ethics Committee of the Azienda Ospedaliero-Universitaria of Pisa approved all recruitment and assessment procedures. Eligible subjects provided written informed consent, after receiving a complete description of the study and having the opportunity to ask questions. Subjects were not paid for their participation according to Italian legislation.

### Measures

Assessment procedures included the SCID-5-RV [21], the Adult Autism Subthreshold Spectrum (AdAS Spectrum), and the Catatonia Spectrum (CS). Questionnaire were carried out by psychiatrists who were trained and certified in the use of the study instruments.

#### The Adult Autism Subthreshold Spectrum

AdAS Spectrum is a questionnaire devised to assess not only full-blown ASD, but also the broader spectrum of subthreshold autism, in subjects with normal intelligence and without language impairment across the lifetime. It allows evaluating a wide area of clinical and nonclinical traits, typical and atypical manifestations, including some gender-specific features. The instrument is composed of dichotomous questions, grouped into seven domains: childhood/adolescence, verbal communication, nonverbal communication, empathy, inflexibility and adherence to routine, restricted interests and rumination, and hyper–hypo reactivity to sensory input. In the validation study [1], the AdAS Spectrum questionnaire demonstrated an excellent reliability and a strong convergent validity with other scales employed in this field, such as the Autism-Spectrum Quotient Test [22] and the *e* Ritvo Autism and Asperger Diagnostic Scale 14-item version [23]. The AdAS Spectrum has been used, in recent years, within numerous studies focusing on the autism spectrum both in clinical and in nonclinical settings [24–33].

#### The Catatonia Spectrum

CS is a self-assessment questionnaire that investigates nuclear, subthreshold, atypical, and partial manifestations of the CS, referred to across the lifespan, divided into domains, and explored with a set of questions. The CS consists of 74 items and is divided into eight domains: (a) psychomotor activity (stupor); (b) verbal response (mutism); (c) repetitive movements (stereotypes); (d) artificial expressions and actions (mannerisms); (e) oppositivity or poor response to stimuli (negativism); (f) response to instructions given from outside (automatic obedience); (g) automatisms; (h) impulsivity. For each item, there is a dichotomous answer “Yes” and “No.”

In the validation study [34], the CS questionnaire demonstrated excellent internal consistency and test–retest reliability and strong convergent validity with alternative dimensional measures of catatonia, such as the Bush-Francis Catatonia Rating Scale and the Bush-Francis Catatonia Screening Instrument [35].

### Statistical analysis

Analyses were performed using Statistical Package for the Social Sciences (SPSS) version 26.0 [36].

Relationships between AdAS Spectrum and CS total scores within the total sample, gender and diagnostic subgroups were studied by calculating Pearson’s correlation coefficients and performing linear regression analyses.

Multiple linear regression was used to identify the AdAS Spectrum domains most strongly correlated with the total CS score.

To study catatonic symptoms in groups with different levels of autism severity, we divided the sample on the basis of the two cut-off points provided by the AdAS Spectrum on its total score [37], obtaining three subgroups: participants with ASD (ASD group); participants with subthreshold autistic traits (AT group); and participants without autistic traits (Non-AT group) and comparing the three obtained groups on CS total score within each the four diagnostic subgroups; for this purpose, we used a one-way analysis of variance (ANOVA) followed by Bonferroni’s test for *post hoc* comparisons. Finally, to complete the study of the relationship between the three ASD, AT, and Non-AT groups and the four diagnostic groups, contingency tables and the Chi-Square test were used.

## Results

The four diagnostic groups were sufficiently homogeneous in terms of age and gender.

The catatonia group included subjects with a mean age of 40.45 years (±11.85) and consisted of 34 (39.5%) males and 52 (60.5%) females. The MDD subjects had a mean age of 40.74 (±11.46) years and consisted of 36 (34.6%) males and 68 (56.7%) females. The group of BDP subjects had a mean age of 40.54 (±13.599) years and consisted of 31 (38.3%) males and 50 (61.7%) females. The group of HC subjects had a mean age of 37.71 (±11.02) years and consisted of 38 (36.2%) males and 67 (63.8%) females.

A statistically significant positive correlation was found between AdAS Spectrum and CS total score within the total sample (*r* = 0.762; *p* < 0.001), the gender subgroups (*r* = 0.740; *p* < 0.001 in males; *r* = 0.776; *p* < 0.001 in females) and in the diagnostic categories (Table 1).

**Table 1.** Correlations between total Adult Autism Subthreshold (AdAS) and Catatonia Spectrum (CS) scores in the diagnostic groups.

Total CS score				
	HC	MDD	BPD	CTN
Total AdAS score	*r* = 0.771	*r* = 0.691	*r* = 0.711	*r* = 0.637
	*p* < 0.001	*p* < 0.001	*p* < 0.001	*p* < 0.001

*Abbreviations:* BPD, borderline personality disorder; HC, helthy control; MDD, major depressive disorder; CTN, catatonia.

Table 2 reports the regression coefficients and their confidence intervals obtained considering total AdAS Spectrum score and CS total score, respectively, as independent and dependent variables in seven regression analyses conducted within the total sample, the male and female gender subgroups, and the four diagnostic groups. All the regression coefficients appeared to be significant and positive underlining a strong general relation between autistic and catatonic symptomatology.

**Table 2.** Seven simple linear regressions [dependent variable: Catatonia Spectrum (CS) total score; predictors: total Adult Autism Subthreshold (AdAS) Spectrum score] related to seven different subgroups, with related regression coefficients (*B*) and standard error (*SE*), significance (*p*), and confidence interval (CI).

Total AdAS score	*B* (*SE*)	*p*	CI (95%)
Total sample	0.414 (0.016)	<0.001	0.383; 0.445
Males	0.404 (0.26)	<0.001	0.352; 0.456
Females	0.42 (0.019)	<0.001	0.382; 0.458
HC	0.438 (0.029)	<0.001	0.380; 0.495
MDD	0.367 (0.032)	<0.001	0.304; 0.430
BPD	0.388 (0.038)	<0.001	0.313; 0.463
CTN	0.383 (0.045)	<0.001	0.293; 0.473

*Abbreviations:* BPD, borderline personality disorder; HC, healthy control; MDD, major depressive disorder; CTN, catatonia.

**Figure 1.**
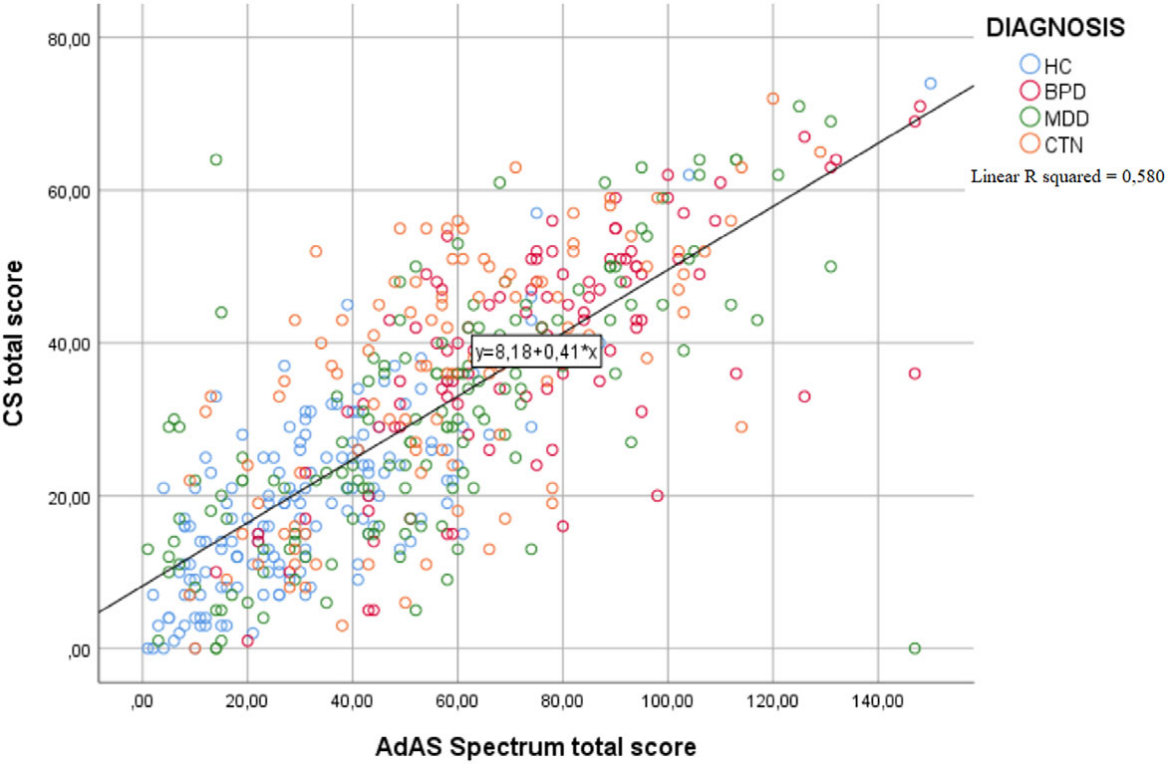
Scatter graph showing the correlation between Adult Autism Subthreshold (AdAS) Spectrum and Catatonia Spectrum (CS) total score within the four diagnostic groups, represented by the plotter points.

In order to get a clearer view of the correlation between total AdAS Spectrum score and total CS score within the different diagnostic groups, we constructed a scatter graph in which the differently colored plotter points represent the subjects belonging to the four diagnostic subgroups (Figure 1).

Table 3 reports the results of the multiple linear regression utilizing the total CS score as a dependent variable and the AdAS Spectrum domain scores as an independent variable. The AdAS Spectrum domains found to be significantly and strongly correlated with the total CS score were: *hyper–hypo reactivity to sensory input, verbal communication, nonverbal communication, restricted interests and rumination, and inflexibility and adherence to routine.*

**Table 3.** Multiple linear regression [dependent variable: total Catatonia Spectrum (CS) score; independent variables: Adult Autism Subthreshold (AdAS) Spectrum domain scores] with related regression coefficients (*B*) and standard error (*SE*), significance (*p*), and confidence interval (CI).

AdAS Spectrum domain scores	*B* (*SE*)	*p*	CI (95%)
Childhood/adolescence	0.151 (0.141)	0.284	−1.25; 0.427
Verbal communication	0.671 (0.221)	**0.002**	0.237; 1.104
Nonverbal communication	0.58 (0.149)	**<0.001**	0.286; 0.874
Empathy	−0.134 (0.215)	0.535	−0.556; 0.289
Inflexibility and adherence to routine	0.252 (0.109)	**0.022**	0.037; 0.466
Restricted interests and rumination	0.536 (0.168)	**0001**	0.207; 0.866
Hyper–hypo reactivity to sensory input	0.798 (0.182)	**<0.001**	0.440; 1.155

*Note:* Adjusted *R*^2^ = 0.589.

p value < 0,05

The ASD, AT, and Non-AT groups were found to be distributed across all diagnostic groups, and in particular, the ASD group was most represented in patients with BPD (56.5%) and in second place in patients with Catatonia (31.1%), the AT group in patients with Catatonia (31.1%), and the non-AT group in healthy controls (75.6%).

**Figure 2.**
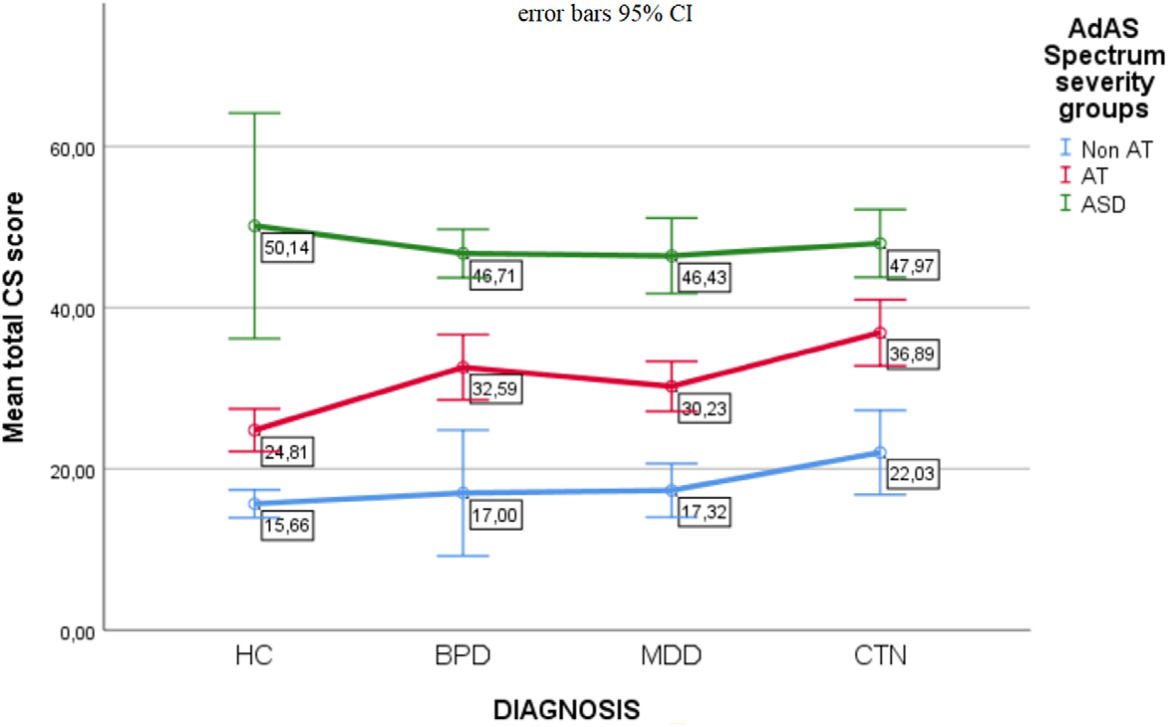
Comparison of the three autism severity groups based on the total Catatonia Spectrum (CS) score. Each of the three lines represents an autism severity group. The points on each line correspond to the average total CS scores in the four diagnostic groups.

Table 4 shows the results of the ANOVA comparing the three autism severity groups based on the CS total score within each diagnostic subgroup. The CS score increased significantly from the non-AT group to the AT group to the ASD group. In other words, the greater the severity of autism, thus the higher the AdAS Spectrum score, the higher the CS score. See also Figure 2 for greater clarity.

**Table 4.** Comparison of the three autistic severity groups based on the Catatonia Spectrum (CS) total score within each diagnostic subgroups.

Diagnostic subgroups	Non-AT (*a*) (mean ± SD)	AT (*b*) (mean ± SD)	ASD (*c*) (mean ± SD)	*F*	*p*	*Post-hoc* comparisons (*p* < 0.05)
HC	15.66 ± 9.45	24.81 ± 7.19	50.14 ± 15.14	52.33	<0.001	*a* < *b* < *c*
MDD	17.32 ± 11.66	30.23 ± 11.64	46.43 ± 14.66	59.88	<0.001	*a* < *b* < *c*
BPD	17.00 ± 10.15	32.59 ± 12.12	47.71 ± 11.49	34.75	<0.001	*a* < *b* < *c*
CTN	22.03 ± 13.77	36.89 ± 13.48	47.97 ± 11.85	30.45	<0.001	*a* < *b* < *c*

*Abbreviations:* ASD, autism spectrum disorder; AT, autistic traits; Non-AT, non autistic traits; BPD, borderline personality disorder; HC, healthy control; MDD, major depressive disorder; CTN, catatonia.

## Discussion

The results of the present study suggest a strong correlation between autism spectrum (autistic traits and ASD signs, symptoms, and behavioral manifestation) and catatonic spectrum (nuclear, subthreshold, atypical, and partial manifestations), in agreement with previous literature regarding the correlation between ASD and catatonia.

The finding of higher CS scores as autism severity increases supports the hypothesis that catatonia could be considered as a neurodevelopmental disorder, in particular a late and potentially preventable form of autism [6, 9]. In this regard, a recent study reported the case of a young child in whom the onset of catatonia, occurring after trauma and against the backdrop of a stressful family environment, led to the diagnosis of an underlying ASD [38].

The autism spectrum features that, in the present study, were found to be most correlated with catatonic spectrum manifestations are, in order of importance, hypo/hypersensitivity to sensorial stimuli, difficulties in verbal and nonverbal communication, mental rumination, and repetitive behaviors. This finding could be explained by considering the role of trauma in the psychopathological trajectory originating from an autism spectrum-derived vulnerability and culminating in catatonic spectrum manifestations.

With regard to the phenomenology of catatonia, Shorter and Fink, in their book The Madness of Fear [39], used a historical analysis to illustrate that catatonia has often been linked to and can be driven by fear. Acute emotional states, especially fear, have long been suggested as a crucial component of catatonia. A recent study reported that half of the patients with phenomenological descriptions reported significant distress at the time of catatonia, and more than one-third of patients explicitly reported fear [40]. The role of trauma in influencing a vulnerable individual to develop catatonic symptoms was explored by a recent study, in which a meaningful correlation was found between the Bush-Francis Catatonia Scale and the Adverse Childhood Experience questionnaire [41]. Based on the same study, there appear to be two constructs of conditioning that predispose a subject to catatonic symptoms in response to traumatic events: external conditioning (e.g., the external environment) and internal conditioning (e.g., flashbacks or nightmares after a traumatic experience). In relation to vulnerability to trauma, clinical and scientific data show an increased risk of adverse events and trauma in individuals with ASD. It has been proposed that individuals with ASD may be more vulnerable to traumatic stress reactions due to difficulties with language comprehension, information processing, emotion regulation impairments, and increased levels of social isolation [42, 43]. In addition, aspects of cognitive processes correlated with ASD, such as repetitive and perseverative tendencies, could contribute to the experience of trauma. In brief, if an experience appears to be particularly salient and perhaps distressing, such as a traumatic event, individuals with ASD may persevere in thoughts and feelings related to that experience, risking re-experiencing the past trauma over and over again [44]. Finally, it has been proposed that the core symptoms of ASD may lead to chronic exposure to daily ASD-related stressors from sensory sensitivity and aversions [45, 46]. In this context, the hypersensitivity typical of autistic individuals could be responsible for an increased intensity of post-traumatic re-experiencing, which is known to be associated with an altered processing of sensations from the external and internal world [47]. This perspective would be in agreement with the so-called “Intense World Theory” according to which, in individuals with autism are characterized by hyper-perception, hyper-attention, hyper-memory, and hyper-emotionality. The progression of the disorder would be driven by over-reactions to experiences that lead the brain to a state of hyper-preference and over-selectivity, which becomes more extreme with each new experience and can be especially accelerated by particular emotional experiences and trauma [48]. Considering these issues, the correlation between autistic traits and catatonic manifestations could be mediated by the vulnerability to trauma derived from particular dimensions of the autistic spectrum, such as mental rumination, communication impairment, and, especially, altered sensitivity to external and internal sensorial stimuli.

## Conclusions

The results of the present study show a strong correlation between the autism spectrum (autistic traits and symptoms of ASD) and the manifestations of CS.

The autism spectrum domains found to be most closely related to CS are, in order of importance, hyper–hypo reactivity to sensory input, verbal communication, nonverbal communication, restricted interests and rumination, and inflexibility and adherence to routine.

## Data Availability

The data that support the findings are available from the corresponding author upon reasonable request.
